# Access and Barriers to Immunization in West Bengal, India: Quality Matters

**DOI:** 10.3329/jhpn.v31i4.20050

**Published:** 2013-12

**Authors:** Debjani Barman, Arijita Dutta

**Affiliations:** ^1^Institute of Health Management Research (IIHMR), Nazirabad, PO Ucchepota (besides Heritage Group of Institutes), Kolkata 700 150, India; ^2^Department of Economics, University of Calcutta, India

**Keywords:** Access, Barriers, Immunization, Quality, Indiay Matters

## Abstract

While many studies attempted to evaluate performance of immunization programmes in developing countries by full coverage, there is a growing awareness about the limitations of such evaluation, irrespective of the overall quality of performance. Availability of human resources, equipment, supporting drugs, and training of personnel are considered to be crucial indicators of the quality of immunization programme. Also, maintenance of time schedule has been considered crucial in the context of the quality of immunization. In addition to overall coverage of vaccination, the coverage of immunization given at right time (month-specific) is to be considered with utmost importance. In this paper, District Level Household and Facility Survey-3 (DLHS-3) 2007-2008 data have been used in exploring the quality of immunization in terms of month-specific vaccine coverage and barriers to access inWest Bengal, India. In West Bengal, the month-specific coverage stands badly below 20% but the simple non-month-specific coverage is as high as 75%. Among the demand-side factors, birthplace of the child and religion of the household heads came out as significant predictors while, from the supply-side, availability of male health workers and equipment at the subcentres, were the important determinants for month-specific vaccine coverage. Hence, there should be a vigorous attempt to make more focused planning, keeping in mind the nature of the barriers, for improvement of the month-specific coverage in West Bengal.

## INTRODUCTION

The single-most crucial indicator of the success of immunization programme in any economy is probably the overall coverage of all essential vaccines and/or individual vaccines separately among the general population. While many studies attempted to evaluate performance of immunization programmes in developing countries by full coverage (BCG, three doses of Polio, and three doses of DPT and Measles vaccines) or the dropouts (1-6), there is a growing awareness about the limitations of evaluating the immunization performance solely by its coverage, irrespective of the overall quality of performance (7). The maintenance of time schedule has been considered crucial proxy indicator of the quality of coverage of immunization (7-9). The other possible ways to look into the quality of immunization is to consider the availability of manpower, equipment, and drugs (10-12). The results of studies in Africa and Asia report that the quality of vaccination services still leaves much to be improved in developing countries (12). They found that the principal problems in maintaining the quality in vaccination process are as follows:

- Inadequate supplies, particularly of vaccines, vaccination cards, registration materials, and other drugs
- Lack of providing appropriate information on vaccines, vaccine-preventable diseases, and vaccination schedules in the vaccination sessions
- Poor training facilities for health workers adversely affecting the frequency and regularity of vaccination sessions
- Inaccuracies in the registration of vaccinations.

The current immunization schedule recommended by World Health Organization (WHO) is designed to give effective protection at the earliest possible age (13). Each vaccine dose is scheduled using two main factors. First, it is scheduled for the age when the body's immune system will work the best. Second, it is balanced with the need to provide protection to infants and children at the earliest possible age (14). However, in almost all developing as well as developed countries, there is significant interruption between the recommended and the actual time of immunization, which assumes a more critical role, delaying subsequent immunization process by snowballing effect with an added risk of outright dropout (15). Such delays are also likely to undermine the effectiveness of immunization platforms for implementing integrated child health interventions as currently encouraged in developing countries where a high proportion of infants are born outside hospitals (16). For example, a study in Mexico shows that delaying vaccination by four months could increase the number of preventable deaths by 18% compared to vaccinating at the recommended ages (17). Thus, delays and untimely immunization intervention would enhance cost-effectiveness; vaccines are extremely costly in poor and developing countries with fiscal austerity. Therefore, in addition to overall coverage of vaccination (which is termed henceforth as ‘non-month-specific’), the coverage of immunization given at right time (which is termed henceforth as ‘month-specific*’*) is to be considered with utmost importance. However, till date there is hardly any literature that estimates month-specific coverage in developing countries. Only a few studies done in some developed countries are available. For example, Hull and Macintyre examined the trends and factors associated with infant vaccination in Australia (18). Clark and Sanderson have found that there is wide variation in timeliness of vaccine coverage within and between the 45 low- and middle-income countries (19). Additionally, there is a very few socioeconomic studies to understand the barriers to receiving timely immunization dosage. However, those were done in developed countries (20,21).

The definition of healthcare access that has earned the most unanimous agreement among the researchers describes it as the ‘timely use of services’ as per need (22-25). There are multiple factors that influence such ‘timely use’ or hinder and interfere with people's access to healthcare (23-28). Again, in 2005, universal coverage was defined as access for all to appropriate promotive, preventive, curative and rehabilitative services at an affordable cost by the WHO member states (29). Following these two concepts of access and coverage, we considered coverage of month-specific vaccination as a proxy for access to quality immunization.

Apart from supply-side issues of the availability of resources and manpower, there are certain demand-side characteristics which are proven to be crucial in receiving the quality of immunization at right time. Religion and socioeconomic status of a household, mother's education and employment status, and gender of the child are assumed to play crucial roles in accessing immunization with maximum quality and effectiveness, although quality is generally assumed to be a purely producer's subject. The ability to utilize the services available is often controlled by socioeconomic gradients of healthcare. Hence, it is extremely important to look into the quality of immunization by its month-specific correctness, along with identifying its supply-side constraints and demand-side barriers to access.

The objectives of this paper are: (a) to explore the quality of child immunization in West Bengal, India, both by its supply-side characteristics as well as timeliness of immunization and (b) to find out how far the supply-side factors are responsible for lack of timeliness of immunization dosages received as well as to identify the demand-side barriers to access quality immunization.

West Bengal is a middle-rung performer in health status among all states in India. The state has general non-month-specific vaccination coverage, institutional delivery, with mothers taking antenatal check-up higher than the Indian national average; Infant mortality rate is far lower than the national average. However, the state has one of the highest rural-urban disparities in most of maternal and child healthcare indicators (30).

The main rationale of the entire exercise in this paper is to bring in the nature and causes of vaccination with or without quality as it might call for specific policy implications to improve the cost-efficiency of the entire programme. This is discussed in the final section of policy directions.

## MATERIALS AND METHODS

We have used District Level Household and Facility Survey-3 (DLHS-3) 2007-2008 data for this paper. The District Level Household and Facility Survey is one of the largest demographic and health surveys ever carried out in India, with a sample-size of about 7,00,000 households, covering all the districts of the country. The present DLHS-3 is the third in the series and, as in two earlier rounds, is designed to provide estimates of maternal and child health, family planning, and other reproductive health services (31).

DLHS-3 adopted a multi-stage stratified probability proportion-to-size sampling design. The International Institute for Population Sciences (IIPS) was designated as the nodal agency for carrying out the survey. Bilingual sets of questionnaire in local language and in English, pertaining to household, ever-married women (aged 15-49 years), unmarried women (aged 15-24 years), were used. Separate sets of questionnaire for village and health facilities were used for gathering required information. DLHS covered only four types of government facilities: (a) subcentres, (b) primary health centres, (c) community health centres, and (d) district hospitals.

The household questionnaire collected information on all members and socioeconomic characteristics of the household, assets possessed, number of marriages and deaths in the household since January 2004. The questionnaire on ever-married women contained information on women's characteristics, maternal care, immunization and childcare, contraception and fertility preferences, reproductive health, including knowledge about HIV/AIDS. Each respondent was asked about her last surviving child and the last but one surviving child. The data included child's age, sex, and immunization status. If the immunization card was available and could be seen, the vaccine-wise date, month and year were copied from the card for BCG, Polio 0 (zero dose Polio vaccine), three doses of DPT, three doses of Polio and Measles vaccines and vitamin A. If the respondent could not show the child's immunization card, she was probed about all these vaccines one by one. Next, information on reasons for not vaccinating and on place of vaccination was collected.

The village-related questionnaire contained information on the availability of administrative services, health, education, water and sanitation, electricity, and other facilities. The data were collected from the same sampled villages where the household survey had been conducted.

For the first time, a population-based facility survey has been conducted in DLHS-3. At the district level, all community health centres and the district hospital were covered. Further, all subcentres and primary health centres, which were expected to serve the population of the sampled village, were also covered.

The health facility-related questionnaire contained information on human resources and their training, physical infrastructure (building, water supply, electricity, toilet facility, staff quarters, waste disposal), and on the availability of selected furniture, equipment, and essential drugs. Next, information was collected on services provided in the health facility in terms of beneficiaries. Lastly, information was collected on monitoring and supervision. Fieldwork in West Bengal was conducted during December 2007 to April 2008, gathering information from 22,213 households, 21,878 ever-married women, 725 villages, 688 subcentres, 290 Primary Health Centres, 335 Community Health Centres, and 19 district hospitals.

For this paper, we used data from (a) ever-married women, (b) village, and (c) subcentres. Although the household-specific data were in the separate dataset, a few data, like those on religion, caste, economic status of the household, were also available in the dataset on ever-married women. Data on mother's age, educational status, and occupation were in the dataset on ever-married women. From the village-related questionnaire, we took data on the availability of village-level infrastructure, like availability of electricity and road connectivity. Among the sets of facility survey questionnaire, we considered subcentre because in as many as 82.7% cases, subcentre remained the place of vaccination. In fact, child immunization under the Universal Programme on Immunization is in the job responsibilities of the female health workers or auxiliary nurses and midwives (ANMs) who operate from the subcentre. From the subcentre-related questionnaire, we took data on the availability of human resources, the level of training, availability of equipment and drugs, and the vaccination-related consumables. There was no information about the side-effects of the immunization for children.

A child is considered fully vaccinated when she/he received a vaccination against tuberculosis (BCG), three doses of the diphtheria, whooping cough (pertussis) and tetanus (DPT) vaccine; three doses of the poliomyelitis (Polio) vaccine; and one dose of the Measles vaccine by the age of 12 months (Table 1). As per the immunization schedule followed in India, a child should first receive BCG at birth; there should be a gap of one month in between the three doses of DPT, and similar gap should be maintained in case of Polio vaccine as well (32). From BCG to Measles vaccine, there should be a gap of 9 months and, from DPT3 to Measles vaccine, the recommended gap is 5.5 months.

**Table 1.** Child immunization schedule

Name of vaccine	Scheduled time in month	Scheduled time in week
BCG	At birth	0
DPT	1.5, 2.5, 3.5	6, 10, 14
Polio	1.5, 2.5, 3.5	6, 10, 14
Measles	9	36
Source: GOI, http://cbhidghs.nic.in/hii2003/12.01.htm (accessed on 19 June 2011)		

From the questionnaire on ever-married women, the non-month-specific vaccine coverage included both mothers’ records as well as information from vaccination card. However, for month-specific cases where one needs to calculate the actual time of taking the vaccine, only the children for whom the vaccine cards were available and could be actually seen were incorporated.

Hence, while the non-month-specific immunization status can be divided into three categories—full, partial, and no—month-specific cases were divided into two categories—full and partial. As month-specific vaccine coverage was calculated from the vaccination cards only, there was not any case of ‘no’ coverage.

As different information was available from three different datasets, we merged the three datasets and prepared a single data file. All these individual datasets actually have the similar coding for state, district, and village. Therefore, we merged these different datasets and made a single one, using these codes. Lastly, given the definition of full immunization for its calculation, we considered only those children who were aged between 12 and 23 months. The merged dataset had 1,636 children.

In this merged dataset, we generated variables for each vaccine, showing the age in month at which the individual vaccines were received, based on the information collected from the immunization card. For calculating the time interval in month for each of these vaccines, we deducted the month at which a particular vaccine was received from the prior one, which a child should receive as per the schedule. Here, we considered six intervals: (i) in between DPT1 and DPT2, (ii) in between Polio1 and Polio2, (iii) in between DPT2 and DPT3, (iv) in between Polio2 and Polio3, (v) in between BCG and Measles vaccine, and (vi) in between DPT3 and Measles vaccine. For gaining further insight into the problem of non-month and month-specific immunization coverage, we divided the immunization coverage in two parts: Polio vaccine coverage and other non-Polio vaccines coverage. While the first one included three oral vaccines against polio, the next one comprised vaccine against childhood tuberculosis (BCG), three shots for diphtheria, pertusiss, and tetanus (DPT), and one shot for measles. The rationale of dividing the total vaccines in two categories is that, for both types, we need separate set of infrastructure altogether. While the Polio vaccine is oral, non-Polio vaccines are injectable and, hence, need specific training, infrastructure, and awareness for success.

In the paper, we divided all the districts of West Bengal into two groups, namely poor-performing Health System Development Initiative (HSDI) districts and relatively better-performing Non-HSDI districts. In six districts (Murshidabad, Bankura, Puruliya, Maldah, Uttar Dinajpur, and Birbhum) the DFID-funded HSDI programme was launched in 2004 for improvement in infrastructure. The rest of the districts were taken together in non-HSDI category (33).

To understand the present infrastructure status and quality of human resources in terms of training received at the subcentres in these two groups of districts, we considered the variables from the supply-side, which are supposed to be crucial for delivery of services with precision. As per literature (12) and available data in DLHS-3, we considered the availability of Paracetamol drug (used for reducing side-effects in non-polio cases), disposable syringes, vaccine carriers, share of auxiliary nurse-midwives (ANMs) with immunization training, and share of subcentres visited by senior officers one month before survey. While the last one indicates the presence of monitoring mechanism, the one before that stands for the technical competence of the frontline health workers who perform the duty in immunization sessions in most cases.

Given the study objective, we considered month-specific vaccine coverage as dependent variable. It was a categorical variable showing whether the child received all the vaccines at right month (or month-specific) or not. We considered three different dependent variables: (a) all month-specific vaccines or month-specific full immunization coverage, (b) month-specific Polio vaccines coverage, and (3) month-specific non-Polio vaccines coverage.

Independent variables can be clubbed under two groups: (a) demand and (b) supply. From demand-side, we considered variables on background characteristics, like gender of the child, place of delivery, birth order, education and employment status of the mother, and religion and economic status of the household. From supply-side, we took village electrification status and availability of male health worker and constructed two indices—one for drug and another for equipment, using Principal Component Analysis (PCA). For the economic status of the household, we divided the wealth index score already given in the dataset on ever-married women into five quintiles.

Under the equipment index, availability of instrument sterilizer, auto-disposable syringe, hub cutter, B.P instrument, stethoscope, weighing machine both for infants and adults, haemoglobinometer, foetoscope, SIMS speculum, IUD insertion kit, and vaccine carrier were included (all those available in DLHS-3). Scale reliability coefficient for equipment index is 0.81. The score was divided into five quintiles, the poorest one was considered reference category, and the rest four categories were clubbed together (34).

**Figure:**
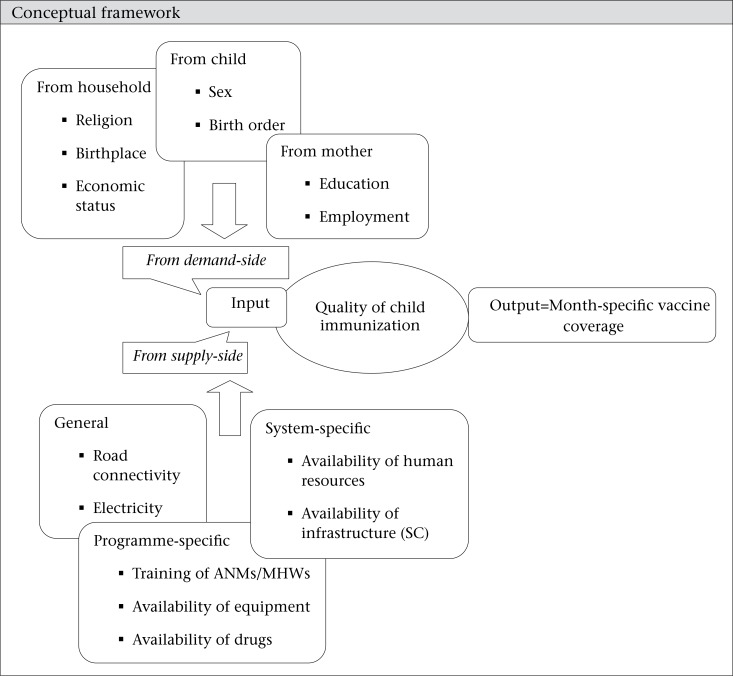
Conceptual framework

The drug index was also constructed using the same method. Under the drug index, drug kit A, drug kit B, IFA tablet, vitamin A solution, ORS packet, injection Gentamycin, Magnesium sulphate, Ampcilin capsule, Metronidazole, Misoprostol, and Paracetamol tablets were incorporated, if these were available in the SC and were not out of stock for more than 10 days during the last one month [Scale reliability coefficient for drug index is 0.84]. Similar method was followed for creating the categories.

Statistical software STATA (StataIC 10) was used for data analysis.

Given the binary dependent variable, we used logistic regression to identify the barriers to accessing the month-specific vaccine coverage. Logistic regression model is a discrete regression model. When the dependent variable assumes discrete values, we called it a discrete regression model. The simplest of these models has a binary dependent variable ‘y’, which can assume only two values. Usage of such a model is very common in social science and medical sciences where the outcome variable is binary in nature. More importantly, one is likely to have categorical variable when one is measuring an attribute. For example, in the present case, we considered the month-specific immunization coverage as dependent variable where a child can either be fully immunized or be partially immunized.

## RESULTS

### A time interval in between the vaccines and month-specific vaccine coverage

From DLHS-3 data, we found that the recommended intervals in between each vaccine were not maintained in West Bengal (Table 2). The average interval between DPT1 and DPT2 remained 1.6 months while the same in between DPT2 and DPT3 was 1.8 months. While the average interval exceeded the recommended interval in between three dosages of DPT and BCG to Measles vaccine, the same in between DPT3 and Measles vaccine was less than the recommended one. The shortest interval in all these six cases remained zero.

**Figure 1.**
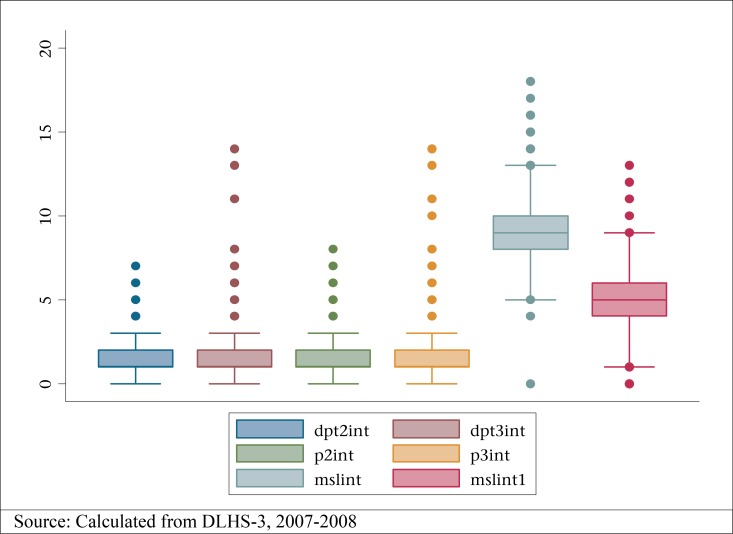
Box plots of individual vaccine intervals

However, looking at the median, mean, and interquartile ranges, it becomes clear that there were several outliers to the entire month-specific data. Figure 1 shows the presence of outliers within the DPT2 and DPT3 gap.

Continuing with the non-month-specific vaccine coverage, we found that the non-month-specific full immunization coverage was 75.9% while that for month-specific coverage was only 16.4% (Table 3). We found that the proportion of children who received all the month-specific non-Polio vaccines was only 17% while that of children who received all the non-month-specific non-Polio vaccines was as high as 76.9%.

### Status at the subcentres of HSDI, non-HSDI districts, and West Bengal state average

The availability of ANMs with immunization training was less in HSDI group of districts compared to non-HSDI group (Table 4). Vaccine carrier was available in 8% SCs while it was not available in 3% SCs in non-HSDI group. In both these groups, auto-disposable syringe was available in as much as 97% SCs. In terms of availability of Paracetamol, SCs under HSDI group marginally lagged behind their counterparts. There was a difference of 5 percentage points in between SCs from HSDI and non-HSDI group of districts in terms of percentage of SCs having visit by monitoring staff during the last month prior to the survey. It was less in HSDI group. Male health workers were available in 37% SCs for HSDI group and in 43% SCs for non-HSDI group.

### Socioeconomic correlates: a demand-side story of immunization with quality

Table 5 shows the share of demographic, social and economic categories with month-specific coverage of Polio and non-Polio vaccines. In the dataset, 1,338 children were with vaccination card. Out of those children, most were of the second birth order. More male children received all the month-specific doses of Polio and non-Polio vaccines compared to female children. In both the cases, when the child was delivered at home, month-specific immunization coverage was lower compared to that of child delivered at any institution. Month-specific immunization coverage was higher among employed mothers compared to unemployed mothers. Hindu children had higher month-specific coverage for both the vaccines. Immunization coverage also improved with higher economic quintile for complete dosages of both types of vaccinations. At the SC level, percentage of complete Polio and non-Polio vaccinations improved with the availability of male health workers and health workers’ visit to SCs.

**Table 2.** Recommended and average interval across the different dosages of vaccines as per card only, West Bengal, 2007-2008

Name of vaccine	Recommended interval in month	Shortest interval in month	Longest interval in month	Average interval in month	Median*
DPT1-DPT2 (dpt2int)	1	0	19	1.6	1 (1)
DPT2-DPT3 (dpt3int)	1	0	16	1.8	1 (1)
Polio1-Polio2 (p2int)	1	0	14	1.6	1 (1)
Polio2-Polio3 (p3int)	1	0	18	1.8	1 (1)
BCG-Measles (mslint)	9	0	20	9.1	9 (2)
DPT3-Measles (mslint1)	5.5	0	13	4.5	5 (2)

*Interquartile range in parentheses; Source: Calculated from DLHS-3, 2007-2008

**Table 3.** Month-specific and non-month-specific vaccine coverage (%)

Type of vaccine	Non-month-specific†	Month specific‡
All vaccines	75.9	16.4
Polio (3 Polio doses)	84.0	25.4
Non-Polio (BCG, 3 doses of DPT and Measles vaccine)	76.9	17.0

†Out of total 1,636 children;

‡Out of 1,338 children with vaccine card; Source: Calculated from DLHS-3, 2007-2008

**Table 4.** Availability of supply-side features in all subcentres covered

Indicator	All districts	HSDI districts	Non-HSDI districts
ANMs with immunization training	70.2	69.6	71.9
Vaccine carrier available	93.9	92.2	96.5
Auto-disposable syringe available	96.4	97.0	97.0
Paracetamol available on the day of survey	86.6	87.8	85.7
Any monitoring staff visited the SC during the last month	64.5	62.0	67.2
Availability of male health workers	39.0	36.6	42.5

Source: Calculated from DLHS-3, 2007-2008

**Table 5.** Background characteristics of children in terms of month-specific immunization coverage

Variable	Total	Month-specific Polio		Month-specific non-Polio	
		Partial	Full	Partial	Full
	1,338	998	340	1,110	228
Characteristics of children					
Gender of the child: Male	695 (51.9)	511 (73.5)	184 (26.5)	577 (83.0)	118 (17.0)
Female	643 (48.1)	487 (75.7)	156 (24.3)	533 (82.9)	110 (17.1)
Birth order of the child	2.0 (1.3)	1.8 (1 .0)	2.1 (1.3)	1.7 (0.9)	2.1 (1.3)
Place of delivery: Home	704 (52.6)	571 (81.1)	133 (18.9)	630 (89.5)	74 (10.5)
Institution	634 (47.4)	427 (67.4)	207 (32.6)	480 (75.7)	154 (24.3)
Mother's characteristics					
Mother's employment: Unemployed	949 (70.9)	702 (74.0)	247 (26.0)	775 (81.7)	174 (18.3)
Employed	389 (29.1)	296 (76.1)	93 (23.9)	335 (86.1)	54 (13.9)
Mother's education: Non-literate	485 (36.2)	399 (82.3)	86 (17.7)	438 (90.3)	47 (9.7)
Up to primary (Class IV)	284 (21.2)	214 (75.4)	70 (24.7)	247 (87.0)	37 (13.0)
Up to middle school (Class VIII)	370 (27.6)	263 (71.1)	107 (28.9)	290 (78.4)	80 (21.6)
Class IX and above	199 (14.9)	122 (61.3)	77 (38.7)	135 (67.8)	64 (32.2)
Religion: Hindu and others	915 (68.4)	667 (70.7)	276 (29.3)	747 (79.2)	196 (20.8)
Muslim	395 (29.5)	331 (83.8)	64 (16.2)	363 (91.9)	32 (8.1)
Household characteristics					
Quintile: Poorest	291 (22.6)	235 (80.8)	56 (19.2)	260 (89.4)	31 (10.7)
Poorer	286 (22.2)	224 (78.3)	62 (21.7)	251 (87.8)	35 (12.2)
Middle	262 (20.4)	193 (73.7)	69 (26.3)	218 (83.2)	44 (16.8)
Richer	266 (20.7)	195 (73.3)	71 (26.7)	211 (79.3)	55 (20.7)
Richest	182 (14.1)	110 (60.4)	72 (39.6)	123 (67.6)	59 (32.4)
Village characteristics					
Village electrified: No	302 (23.9)	223 (73.8)	79 (26.2)	251 (83.1)	51 (16.9)
Yes	957 (76.0)	719 (75.1)	238 (24.9)	797 (83.3)	160 (16.7)
Subcentre characteristics					
Male health worker: No	701 (60.1)	539 (76.9)	162 (23.1)	594 (84.7)	107 (15.3)
Yes	466 (40.0)	329 (70.6)	137 (29.4)	372 (79.8)	94 (20.2)
Equipment index: Poorest	223 (19.2)	186 (83.4)	37 (16.6)	204 (91.5)	19 (8.5)
2nd-5th quintiles	940 (80.8)	678 (72.1)	262 (27.9)	758 (80.6)	182 (19.4)
Essential drug index: Poorest	243 (21.5)	178 (73.3)	65 (26.8)	189 (77.8)	54 (22.2)
2nd-5th quintiles	888 (78.5)	667 (75.1)	221 (24.9)	750 (84.5)	138 (15.5)
During last month SC visited by MO/LHV/MHW: No	409 (35.1)	312 (76.3)	97 (23.7)	349 (85.3)	60 (14.7)
Yes	758 (64.9)	556 (73.4)	202 (26.7)	617 (81.4)	141 (18.6)

Source: Calculated from DLHS-3, 2007-2008

Table 6 represents the results of logistic regressions for month-specific Polio, non-Polio and full vaccine coverage. Among the characteristics of children, gender of the child and birth order did not come out as significant predictor for any of the month-specific vaccine coverage. The children who were delivered at any institution were 1.2 times (95% CI 0.9-1.7) more likely to have all the month-specific coverage for Polio vaccines. The same was 1.8 for month-specific non-Polio (95% CI 1.1-2.4) and full immunization (95% CI 1.1-2.4) coverage. Mother's education came out as a significant predictor for the month-specific vaccine coverage but only when she was educated up to middle school. If the mother was employed, the child was less likely to receive month-specific vaccines; however, it came out as significant only in the case of month-specific full vaccine coverage (OR 0.7, 95% CI 0.4-1.0).

**Table 6.** Odds of child being fully immunized by month-specific Polio and non-Polio cases

Variable	Month-specific Polio coverage	Month-specific non-Polio coverage	Month-specific full coverage
Child characteristics			
Gender of the child: Male (ref.)			
Female	0.81 (0.6-1.1)	1.15 (0.8-1.6)	1.08 (0.8-1.5)
Birth order of the child	1.19 (0.9-1.7)	1.04 (0.7-1.5)	1.02 (0.7-1.5)
Place of delivery: Home (ref.)			
Institution	1.21 (0.9-1.7)	1.63 (1.1-2.4)	1.6 (1.1-2.4)
Mother's characteristics			
Mother's employment: Unemployed (ref.)			
Employed	0.85 (0.6-1.2)	0.73 (0.5-1.1)	0.66 (0.4-1.0)
Mother's education: Non-literate (ref.)			
Up to primary (Class IV)	1.09 (0.7-1.7)	1.01 (0.6-1.8)	0.95 (0.5-1.7)
Up to middle school (Class VIII)	1.45 (0.9-2.3)	1.53 (0.9-2.6)	1.55 (0.9-2.6)
Class IX and above	1 (0.6-1.8)	1.19 (0.6-2.3)	1.20 (0.6-2.3)
Religion: Hindu (ref.)			
Muslim	0.39 (0.2-0.7)	0.52 (0.3-1.0)	0.52 (0.3-1.1)
Household characteristics			
Quintile: Poorest (ref.)			
Poorer	1.04 (0.6-1.7)	0.92 (0.5-1.6)	0.96 (0.5-1.7)
Middle	1.05 (0.6-1.8)	0.77 (0.4-1.4)	0.78 (0.4-1.5)
Richer	1.67 (1.0-2.8)	1.2 (0.7-2.2)	1.25 (0.7-2.3)
Richest	1.88 (1.0-3.6)	1.66 (0.8-3.4)	1.72 (0.8-3.5)
Village characteristics			
Village electrified: No (ref.)			
Yes	0.88 (0.6-1.3)	0.88 (0.6-1.3)	0.84 (0.5-1.3)
Subcentre characteristics			
Male health worker: No (ref.)			
Yes	1.74 (1.3-2.4)	1.65 (1.1-2.4)	1.51 (1.0-2.2)
Equipment index: Poor (ref.)			
Better	2.78 (1.8-4.4)	3.39 (1.9-6.0)	3.2 (1.8-5.7)
Essential drug index: Poor (ref.)			
Better	0.73 (0.5-1.0)	0.58 (0.4-0.9)	0.61 (0.4-0.9)
Supervisor visited at least once in last 1 month: No (ref.)			
Yes	0.94 (0.7-1.3)	0.76 (0.5-1.1)	0.85 (0.6-1.3)
District* Religion Interactive Dummy			
HSDI-Hindu	1.15 (0.8-1.7)	1.14 (0.7-1.7)	1.25 (0.8-1.9)
HSDI-Muslim	0.74 (0.3-1.6)	0.43 (0.2-1.1)	0.47 (0.2-1.3)
LR chi2	107.3	107.9	106.5
Prob >chi2	0	0	0
Pseudo R2	0.1	0.12	0.12

Figures in parentheses are 95% CI; Source: Analyzed from DLHS-3, 2007-2008

A Muslim child was less likely to be fully vaccinated compared to Hindu and other counterparts in the case of month-specific Polio (OR 0.4, 95% CI 0.2-0.7), non-Polio (OR 0.5, 95% CI 0.3-1.0), and full vaccination coverage (OR 0.5, 95% CI 0.3-1.1). Household economic status came out as a significant determinant only in the case of month-specific Polio vaccine coverage. With the availability of male health workers, children were 1.7 times more likely to have all the month-specific Polio (95% CI 1.3-2.4) and non-Polio (95% CI 1.1-2.4) vaccine coverage. In the case of full vaccine coverage, it was 1.5 times (95% CI 1.0-2.2) more likely.

When the subcentre belonged to higher quintile in terms of equipment index, the child was more likely to be fully vaccinated in terms of month-specific Polio (OR 2.8, 95% CI 1.8-4.4), non-Polio (OR 3.4, 95% CI 1.9-6.0) and full (OR 3.2, 95% CI 1.8-5.7) vaccine coverage. On the other hand, if the subcentre belonged to higher quintile in terms of drug index, the child was less likely to be fully vaccinated in terms of month-specific Polio (OR 0.7, 95% CI 0.5-1.0), non-Polio (OR 0.6, 95% CI 0.4-0.9) and full (OR 0.6, 95% CI 0.4-0.9) vaccine coverage. With the SC being visited by any medical officer during the last month, the child was less likely to receive month-specific coverage for all types of vaccines. However, it did not come out as a significant predictor.

## DISCUSSION

The subcentre-level infrastructure and human resource availability were poorer for the HSDI group of districts compared to their non-HSDI counterparts with respect to vaccine carrier and male health workers. Greater unavailability of vaccine carriers in HSDI group of districts means that the health workers could not preserve the vaccines at the recommended temperature, and this seriously undermined the issue of quality.

Moreover, questions can be raised in terms of training imparted to ANMs on immunization process. While this was their foremost crucial duty, along with maternal care, limited exposure to specific training for them could seriously truncate their ability to offer quality services to the community. In fact, this training was not provided to ANMs considering the need in relatively backward districts in HSDI group. As most training sessions take place in district headquarters and are organized by district office, the efficiency and eagerness of that office makes a lot of difference. At the same time, the schedules of training sessions are prepared by various sub-divisions of health department at the state capital, and often there are overlapping dates meaning limited applications. As found in this study, the share of ANMs with such training was less in HSDI districts compared to non-HSDI districts.

Availability of male health workers played a crucial role in convincing the male folks of the village about the need of use of maternal and childcare services; they also looked after the general public-health situation in the village. The availability was extremely limited in HSDI districts (many of which were dominated by socially-backward classes where it was difficult to break the traditional views about non- or suboptimal use of childcare services) while, on the whole, male health workers were less in entire West Bengal. Also, the monitoring mechanism appeared to be pretty weak in the state.

Among the characteristics of children, gender of the child and birth order did not appear to be significant predictors in any type of vaccines. This contradicts findings from some of the earlier studies on simple coverage, particularly in South Asia (35,36,37), although findings from some studies do support our observation for simple coverage too (6,38). Place of delivery influenced the vaccine coverage as once the child was delivered at an institution, mother got the opportunity to interact with the public healthcare services and was likely to vaccinate her child.

Mother's education, economic quintile (captured by wealth index class), and ethnicity (captured here by religion) are the oft-quoted socioeconomic gradients of healthcare, which are proven to alter healthcare-seeking behaviour. We found that a child whose mother was employed was almost half less likely to be vaccinated at right time for all the vaccines. This contradicts the idea that mother's employment and, hence, income in their hand not only increases their awareness and also raises the family's expenditure on human development. The explanation of this apparently unexpected phenomenon was sought in terms of the nature of mother's employment. The data show that more than 90% of employed mothers worked in informal sector, and they seldom enjoyed any leave for their children. This created problems for children being taken to nearby subcentres for vaccination by their mothers as their work time clashed with the SC clinic time.

Religion came out as a significant barrier to month-specific vaccine coverage; however, it appeared to be a stronger barrier in the case of month-specific Polio coverage compared to non-Polio vaccine coverage [It was observed that, in the state, majority of Muslims still believed that polio vaccine can affect the child's health detrimentally and, hence, this traditional belief truncated the accessibility of Polio vaccines among them. Special drives are taken to spread the awareness through the Minority Development Boards in most of the districts]. Again, taking the interactive variable between religion and category of districts (HSDI and non-HSDI), we found that being Muslim from HSDI districts created a significant barrier to accessing all immunizations at recommended time compared to a Muslim in non-HSDI districts in the case of non-Polio vaccines, although there was no big difference in the month-specific coverage of Polio vaccines.

The presence of male health workers increased the chance of a child to be fully immunized under both the vaccines. This result is in tune with earlier studies on simple non-month-specific vaccine coverage (31) where it was found that availability of male health workers, who work at the community level, positively influences the households’ decision to take the pregnant women to hospitals for delivery and the overall acceptance of several public-health policies [This does not, however, contradict the usually-observed negative impact of male doctors on healthcare-seeking behaviour of the general population, especially in South Asia. Male health workers work at primary tiers of health facilities and can aware the people just like the female health workers]. Probably, the male health workers can more effectively persuade the male folks in the village to alter their age-old beliefs and healthcare-seeking behaviour. Unfortunately, this cadre in the Department of Health and Family Welfare under the Government of West Bengal is under the slow process of extinction. The picture is not at all optimistic at the national level too [According to the Rural Health Statistics, Central Bureau of Health Intelligence 2010, India has only 36% of male health workers in position compared to their actual requirements while the figure for West Bengal is 39%].

When the subcentre belonged to higher quintile in terms of equipment index, the child was more likely to be fully vaccinated both by Polio as well as non-Polio vaccines. However, as expected, the unavailability of equipment posed a greater barrier for non-Polio vaccines as all of those vaccines are injectable and, hence, require more infrastructural facilities. Availability of all drugs, however, has an ambiguous impact on the month-specific vaccination.

The role of drugs is considered one of the correlates of month-specific vaccine coverage because these drugs are given to the children in case of possible side-effects, like fever, pain, swelling (12). As there is not much evidence of adverse reaction of oral polio dosage, the non-Polio injections commonly refer to side-effects. The low statistical significance of this variable for Polio vaccination is, thus, perfectly understandable. However, the negative sign of the significant coefficient in non-Polio vaccines calls for a more detailed analysis. Focus group discussions with mothers revealed that whenever drugs were available, frontline workers tended to prescribe it even if it was not necessary (Personal communication at fields in Murshidabad and Hugli districts, 2011-2012). Further, administration of drug dosage repels the mothers for these vaccines.

Monitoring of the SCs by medical officers had no significant impact, rather had negative influence on month-specific vaccine coverage. This probably hints towards an overall failure of monitoring in Indian health system (39), more so for maintaining quality in vaccination system (12).

### Conclusions

The basic story that emerges out of this discussion in the paper is that even if West Bengal fares moderately well in overall coverage of the immunization services, indicators of quality hint towards gross underperformance. The system lacks well-trained manpower and supervision in both types of districts. Although basic infrastructure consisting of equipment and drugs was available in the state, the maintenance of time-schedule was neglected most often. Looking at the barriers to access month-specific immunization, a number of demand-side factors were identified as crucial. Mother's education, household's ethnicity, and economic status were some of the crucial correlates of month-specific immunization. These social gradients need to be corrected with more vigorous attempt of making public health facility accessible in vulnerable districts, along with awareness campaign. So far, the public health facilities do not differ much between the two groups of districts, portraying the philosophy of equality, rather than equity, within the health system *per se*. Similarly, on the supply-side, availability of male health workers and proper equipment at the subcentres can improve the access to month-specific immunization, although possible over-use of drugs creates a barrier. The fact that the probability of month-specific immunization improves if the child is delivered at any hospital recons that the mothers, once using the institutional service window, are more likely to continue its use for her children. However, no specific interest has been shown by the state government to improve the availability of manpower and equipment in the near future. In short, the issue of quality in immunization programme should be immediately addressed at all levels to increase the efficiency of this crucial public health programme. An attempt should be made to introduce the alternative schedule of vaccination designed for dropouts, late starters, and wrong intervals.

## ACKNOWLEDGEMENTS

We acknowledge the scientific support extended by Future Health Systems: Innovations for Equity (www.futurehealthsystems.org), a research programme consortium of researchers from Johns Hopkins Bloomberg School of Public Health (JHSPH), USA; Institute of Development Studies (IDS), UK; International Centre for Diarrhoeal Disease Research, Bangladesh (icdd,b); Institute of Health Management Research (IIHMR), India; China National Health Development Research Center (CNHDRC), China; School of Public Health (SPH), Makerere University College of Health Sciences, Uganda. The views expressed are not necessarily those of FHS partner institutions, the DFID, and the Department of Health and Family Welfare, Government of West Bengal. The authors also want to thank faculties and research associates of the Department of Economics, University of Calcutta, for their valuable comments.
